# Genome-Wide Identification and Characterisation of Wheat *MATE* Genes Reveals Their Roles in Aluminium Tolerance

**DOI:** 10.3390/ijms23084418

**Published:** 2022-04-16

**Authors:** Wenjing Duan, Fengkun Lu, Yue Cui, Junwei Zhang, Xuan Du, Yingkao Hu, Yueming Yan

**Affiliations:** Beijing Key Laboratory of Plant Gene Resources and Biotechnology for Carbon Reduction and Environmental Improvement, College of Life Science, Capital Normal University, Beijing 100048, China; duanwenjings@163.com (W.D.); lfksiwuliu@163.com (F.L.); cy9551@yeah.net (Y.C.); zhangjunwei_22@163.com (J.Z.); duxuan503@163.com (X.D.)

**Keywords:** wheat, *MATE* gene family, molecular evolution, expression profiling, molecular docking, aluminium tolerance

## Abstract

The Multidrug and toxin efflux (*MATE*) gene family plays crucial roles in plant growth and development and response to adverse stresses. This work investigated the structural and evolutionary characteristics, expression profiling and potential functions involved in aluminium (Al) tolerance from a genome-wide level. In total, 211 wheat *MATE* genes were identified, which were classified into four subfamilies and unevenly distributed on chromosomes. Duplication analysis showed that fragments and tandem repeats played the main roles in the amplification of *TaMATEs*, and Type II functional disproportionation had a leading role in the differentiation of *TaMATEs*. *TaMATEs* had abundant Al resistance and environmental stress-related elements, and generally had a high expression level in roots and leaves and in response to Al stress. The 3D structure prediction by AlphaFold and molecular docking showed that six TaMATE proteins localised in the plasmalemma could combine with citrate via amino acids in the citrate exuding motif and other sites, and then transport citrate to soil to form citrate aluminium. Meanwhile, citrate aluminium formed in root cells might be transported to leaves by TaMATEs to deposit in vacuoles, thereby alleviating Al toxicity.

## 1. Introduction

Wheat serves as an important staple food crop for 35% of the world’s population. However, increasing heavy metal pollution, especially Al toxicity, seriously affects crop growth and sustainable food production [1]. About 30% of the world’s ice free land areas belong to acid soils, and only 4.5% of the acid soil area is used for arable crops [2]. Under acidic conditions, Aluminum (Al) is solublised to its ionic form, which shows toxicity to plants [3]. Thus, it has been recognised as a major abiotic stress factor in low pH soils and remains a serious obstacle to sustainable food production worldwide [2,4]. In general, phytotoxic levels of Al hamper plant root growth and lead to small and brittle root systems [5], which is associated with alterations in several physiological processes and biochemical pathways [6]. Therefore, it is highly important to develop crop cultivars with high Al-tolerance in the plant breeding programs.

It is known that the strategies of plants for resisting Al toxicity include external and internal Al detoxification [7]. Al tolerance can either be mediated via the exclusion of citrate from the root apex or via intracellular tolerance of Al transported into the plant symplasm. Among these mechanisms, organic acid anions (OA) with low molecular weight, such as citrate have important functions in the external and internal detoxification of Al in different plant species [8,9]. To date, numerous studies have revealed the role of two major gene families *ALMT* (aluminium activated malate transporter) and *MATE* (multidrug and toxic compound extrusion) in Al tolerance in several plant species that encode membrane proteins and facilitate malate and citrate efflux, respectively [10]. The citrate transporters particularly display varying degrees of constitutive or element responsive (Al-activated) expression and may play a role in the detoxification of Al in the rhizosphere [11]. These organic acids can chelate the Al, and then either protect the roots (chelation in rhizosphere) or cellular components (chelation in the cytosol) from the phytotoxic effects of Al [12]. In particular, the conserved citrate exuding motif (CEM) present in MATEs was found to participate in citrate-binding and transportation for Al chelation [13]. Al induced a thicker mucilage layer around detached border cells, the release of an Al-binding mucilage by border cells could protect root tips from Al-induced cellular damage [14]. Thus, the strong and rapid binding of Al can alter cell wall structural and mechanical properties, at the same time, reducing the Al in the cell [12].

The internal tolerance mechanism is involved in the chelation and detoxification of Al in the symplast with carboxylate anions after it enters the plant. For instance, the buckwheat could accumulate Al to a high level in its leaves when the plant was grown on acid soils [15]. Most of the Al was complexed with Al-citrate (1:1) in *Hydrangea* leaves [16]. Leaf compartmental analysis showed that 80% of the Al in buckwheat leaves was stored in vacuoles as a 1:3 Al-oxalate complex [17]. This internal detoxification mechanism includes Al chelation in the cytosol and subsequent storage of the Al-carboxylate complex in the vacuole. At the same time, an oxidative burst is probably involved in the toxicity of Al in roots and plants react to the increased reactive oxygen species (ROS) [18].

MATE transporters have been found in both prokaryotic and eukaryotic organisms, which exhibit a unique topology [19]. These proteins are present in plants in the form of a transporter gene family containing a large number of genes. To date, some MATE homologous genes in plants have been identified. For example, at least 56, 49 and 138 MATE members are present in Arabidopsis, maize, and *Nicotiana tabacum*, respectively [20,21,22]. In maize, 49 *MATE* genes were divided into seven groups, in which subfamily II and III exhibited differential expression patterns under Al stress conditions [21]. The MATE members were classified into four major clades in *Nicotiana tabacum*, and different NtMATE might show specific functions in the transportation substrate [22]. Besides, MATE involves the regulation of plant development such as the efflux of heterologous substances, accumulation of secondary metabolites alkaloids and flavonoids, transfer of Fe, and signal transduction of plant hormones. In Arabidopsis, the MATE family member *FRD3* was an iron chelator in the root xylem, which is necessary for efficient iron uptake out of the xylem or apoplastic space [23]. *EDS5* was homologous with members of the MATE transporter family, strongly induced by salicylic acid, indicating a possible positive feedback regulation [24]. To date, the identification and functional characterisation of only a few *MATE* genes in wheat have been reported [25,26,27,28]. In-depth investigations on the structural and evolutionary characteristics and their functions in Al tolerance are still lacking.

In this work, we used the newly released genome sequence draft (IWGSC RefSeq v2.1, version 44) to perform a comprehensive genome-wide analysis of wheat *MATE* family genes. Our purpose is to reveal their structural and evolutionary characteristics, expression profiling and potential functions involved in Al stress tolerance. Our results provided new insights into the molecular evolution and functional characteristics of the plant *MATE* gene family, which lay a foundation for the genetic improvement of crop cultivars resistant to Al toxicity.

## 2. Results and Discussion

### 2.1. Genome-Wide Identification and Phylogenetic Analysis of Wheat MATE Gene Family

**Figure 1:**
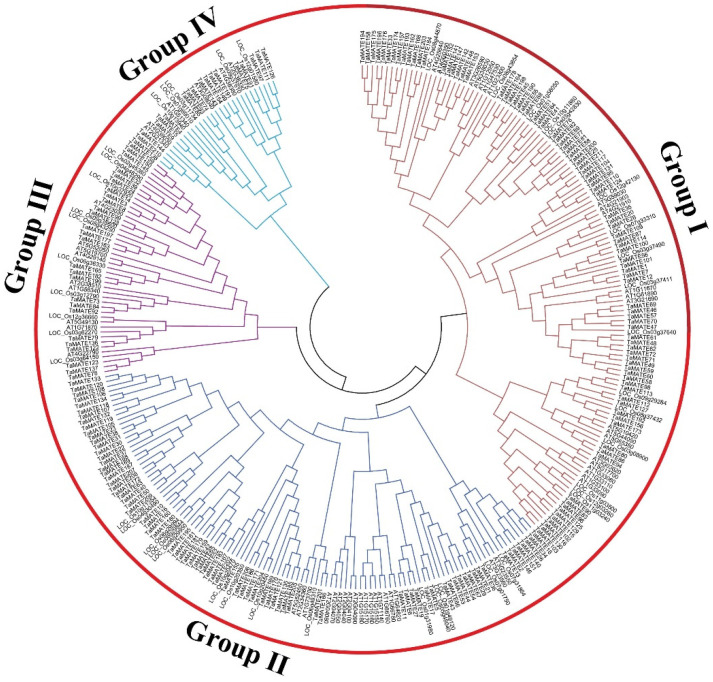
The Bayesian phylogenetic tree of *MATE* (multidrug and toxin efflux) gene family from *Triticum aestivum*, *Arabidopsis thaliana* and *Oryza sativa*. Group I, II, III and IV represent four different subfamilies.

Through blast search against the *Triticum aestivum* genome database from WheatOmics 2.1, a total of 211 wheat *MATE* genes were obtained and named *TaMATE1*–*211*. The results showed that the length of the MATE proteins was 197–642 amino acids with the molecular weight from 21.66 to 66.29 kDa and isoelectric point from 4.98 to 9.78 (Table S1). To obtain more information on the wheat *MATE* gene family, 211 wheat MATE protein sequences were compared with 56 rice and 45 Arabidopsis MATE protein sequences and the phylogenetic tree was reconstructed by the Bayesian method in MEGA (Figure 1). According to the topological structure of the Bayesian tree of three species, all proteins were classified into four subfamilies, named Group I, II, III and IV. As expected, wheat MATE protein family members also had the same four subfamilies, which contained 90, 77, 26 and 18 family members in Group I, II, III and IV, respectively. Since wheat and rice are monocot plants, their *MATE* genes showed a close phylogenetic relationship.

### 2.2. Structural Characterisation of TaMATE Genes

**Figure 2:**
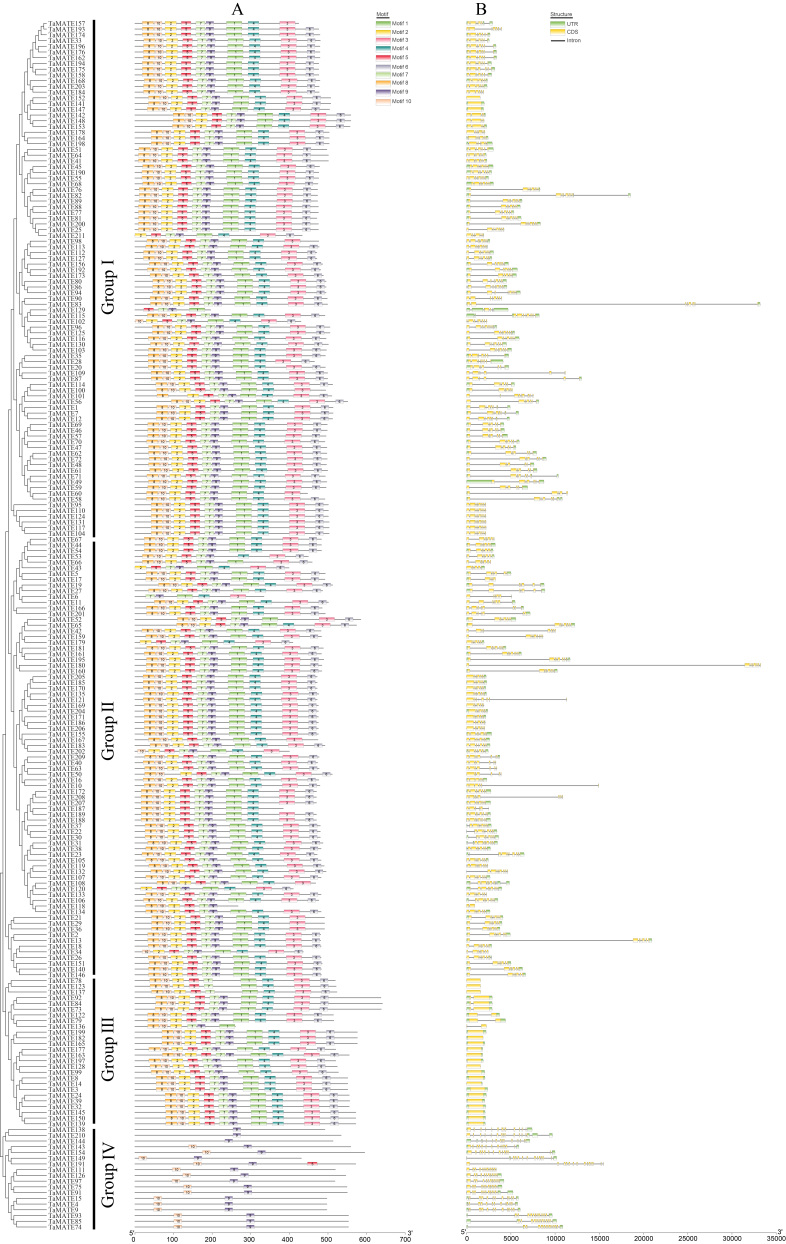
The motif and exon-intron organisation of *MATE* gene family members in wheat. (**A**) Conservative motifs of TaMATE proteins. The motif information was obtained from the MEME webpage and visualised in TBtools. (**B**) Exon-intron structures of *MATE* gene family. The untranslated regions (UTRs) are indicated by green boxes. Yellow boxes represent exons, and the block line represents introns. The sizes of introns and exons can be estimated by the scale at the bottom.

The website of MEME was used to analyze the motif compositions of *TaMATE* genes. As shown in Figure 2A, 10 different motifs were identified among 211 *TaMATE* genes. Both Group I and Group II had the same numbers and shared motifs 7 and 9, in which the majority of members had 9–10 motifs (a few members with four, six and eight motifs). Most of the Group III members had 10 motifs (a few members with six motifs) while the Group IV members only had 1–3 motifs. The motif distribution in different subfamily members displayed certain regularity except for individual members. Motif 9 was possessed by all subfamily members except *TaMATE78*, *TaMATE123* and *TaMATE137*, indicating that motif 9 was highly conserved and might play an important role in maintaining the normal structure and function of TaMATE proteins. Except for a few short TaMATE members, Group I and II had similar motif compositions while Group IV had the least motif species and quantity. Compared with Group I, II and III, all Group IV members only included motifs 5, 9 and 10, indicating that *TaMATE* genes might undergo obvious structural variations and functional disproportionation. In particular, the citrate exuding motif (CEM) in the TaMATEs was found to play an important role in the citrate exclusion to reduce Al toxicity [28]. Interestingly, only six TaMATEs (TaMATE4, TaMATE9, TaMATE15, TaMATE74, TaMATE85 and TaMATE93) from the Group IV subfamily contained CEM. This suggests that these *TaMATE* genes might have undergone evolutionary selection to adapt to Al stress, and the CEM variation might occur after the differentiation of the *TaMATE* gene subfamily.

We further analysed the structure characteristics of 211 *TaMATE* genes in wheat. The members of exon-intron exhibited a large change in different subfamilies (Figure 2B). Group I and B members had the same number of exons, and most members had 6–8 exons (a few members with one, two, three, five, or nine). Group IV members included 1–2 exons while most members in Group III contained 12–14 exons (some members with 10 exons), significantly higher than intron numbers. The intron numbers of different *TaMATE* genes were diverse, except for three and 20 *TaMATE* genes in Group I; Group III had no introns, and the remaining 188 *TaMATE* had different numbers of introns. It is known that the function of genes could be caused by amino acid alterations by substitutions and/or exon-intron structure [29]. Our results indicate that the motif and exon-intron structure in different *TaMATE* subfamily members were diverse while those from the same *TaMATE* subfamily were similar. These suggest that functional differentiation of different *TaMATE* subfamily genes could be accompanied by specific regulatory motifs and exon-introns.

### 2.3. Chromosomal Assignment and Duplication Analysis of TaMATE Genes

**Figure 3:**
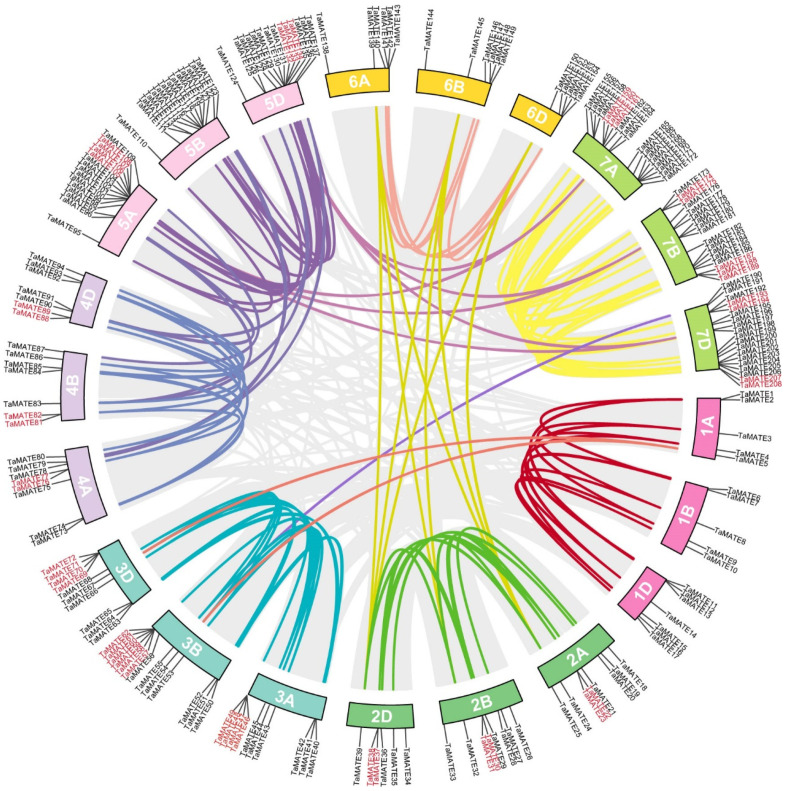
The gene distribution and duplication events of *MATE* gene family members in wheat chromosomes. The different colour lines represent the segmental duplication pairs between the TaMATEs and the gray lines represent the segmental duplication pairs in the whole maize genome. The red marked *TaMATE* genes represent the tandem duplicated genes in the whole wheat genome.

We used MapInspect to analyze the chromosomal distribution of the identified 211 *TaMATE* genes (Figure 3). The results showed that 208 *TaMATE* genes could be assigned to 21 different chromosomes while the location of three genes (*TaMATE209*, *TaMATE210* and *TaMATE211*) was not determined. Among them, 70, 70 and 68 *TaMATE* genes were located, respectively, on the chromosomes A, B and D, indicating their even distribution on the three wheat subgenomes. However, the distribution of TaMATE members on individual chromosomes was uneven. Chromosome 7 had the highest density with 54 TaMATE members from *TaMATE155* to *TaMATE208*, but chromosomes 1 and 6 only contained 17 *TaMATE* genes.

Gene duplication, especially segmental and tandem duplication, is generally considered to be one of the important driving forces in gene family expansion and functional differentiation. As an allohexaploid species, wheat was formed by crossing three different ancestor species, and each wheat gene generally has three homologous loci due to polyploidisation [30]. As shown in Figure 3, 192 *TaMATE* segmental duplication genes were found which consist of two or three copies from the A, B, and D subgenomes and account for approximately 91% of all identified TaMATEs. Interestingly, 211 *TaMATE* genes, 21.33% (45 of 211) originated from tandem duplications. In addition, the tandemly duplicated genes had homologous copies in three subgenomes, indicating that most tandem duplication events occurred before wheat polyploidisation. In addition, the tandem duplication may lead to an intensification of gene expression, for example, in-tandem *MATE* genes showed a high overall expression under the treatment of Al^3+^ tolerance in maize [31]. These results also suggested that *TaMATE* genes were formed by fragment repetition and tandem duplication during the evolution process.

### 2.4. Subcellular Localisation of TaMATE Proteins

**Figure 4:**
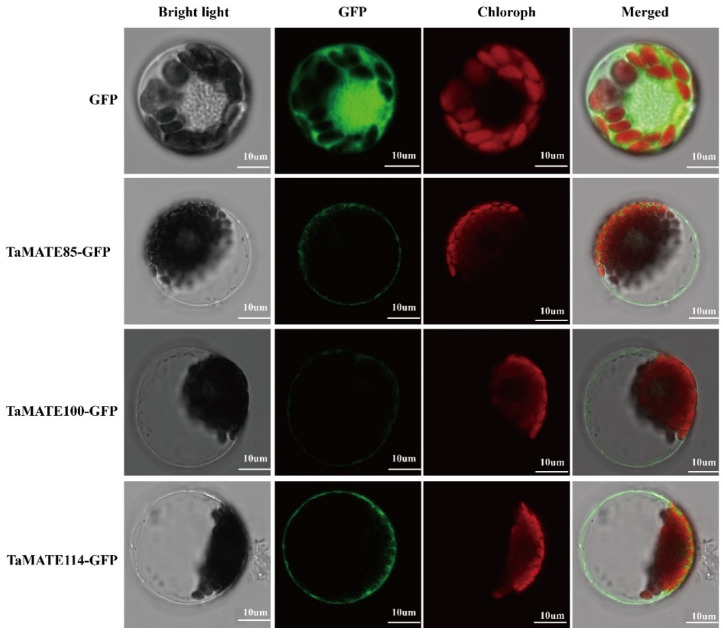
Subcellular localisation of TaMATE85, TaMATE100 and TaMATE114 in wheat leaf protoplast. GFP, GFP fluorescence signal; chloroph, chlorophyll autofluorescence signal; bright light, bright field image; merged, merge of GFP fluorescence signal, chlorophyll autofluorescence signal, and bright field image.

The subcellular localisation of the identified TaMATE proteins was predicted by using WoLF PSORT, Plant-mPLoc, CELLO v.2.5, UniprotKB and TargetP databases (Table S1). Most of the TaMATEs were localised in the plasmalemma (97.16%), followed by the vacuolar membrane (2.84%). We further performed subcellular localisation assay via transient expression in wheat protoplast to verify the reliability of the prediction results. The specific primers were designed (Table S2) and used to amplify the full-length coding sequences of *TaMATE85*, *TaMATE100* and *TaMATE114* genes. Then these genes were cloned onto a 163GFP vector and transiently expressed in wheat protoplast. As shown in Figure 4, the strong green fluorescent signals of three *TaMATE* genes GFP fusion proteins were observed in the plasmalemma, indicating that these genes were located in the plasmalemma. These results were consistent with the website-based predictions (Table S1).

### 2.5. 3D Structure, Functional Disproportionation and Coevolution Analysis of TaMATE Proteins

**Figure 5:**
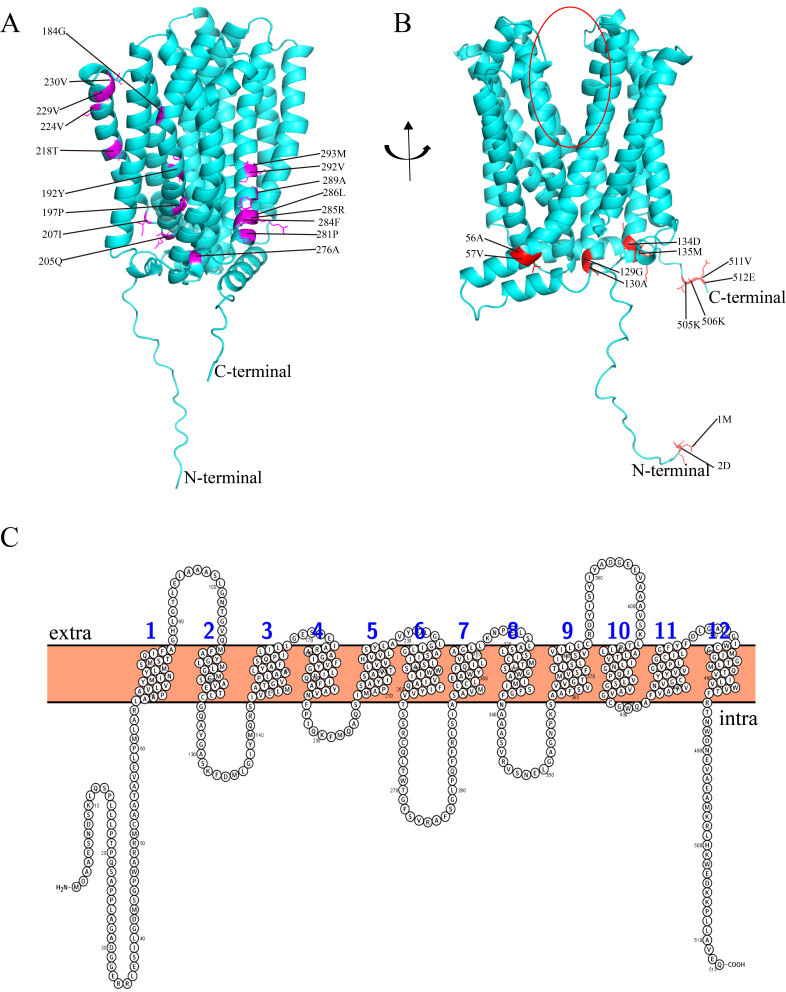
Analysis of 3D structure, functional divergence and coevolution sites in wheat TaMATE1 protein. (**A**) The 3D structure predicted by AlphaFold and 17 key functional divergence sites labelled with purple. (**B**) The 12 sites responsible for coevolution are coloured red. Red circle represents the central cavity of TaMATE. (**C**) The transmembrane helices of TaMATE predicted with Protter webserver; 1–12 represents the 12 transmembrane helices of TaMATE.

TaMATE1 was selected to predict the 3D structure by using AlphaFold (Figure 5). This method produces structure predictions with accuracies approaching and enables the rapid solution of challenging X-ray crystallography and cryo-electron microscopy structure modelling problems, which provides insights into the functions of proteins of currently unknown structures, such as wheat [32,33,34]. The predicted TaMATE 3D model contained 12 α-helices and multiple coils (Figure 5A,B), in which 1–6 and 7–12 α-helices were distributed in N-terminal and C-terminal, respectively. Moreover, the 3D model of TaMATE contained a central cavity located between the N and C domains. The 12 α-helices were also predicted by the Protter website, which belonged to the transmembrane helices (Figure 5C). These transmembrane helices’ structure and central cavity could guarantee the stable function of TaMATE transporters.

Functional disproportionation is a way to increase the rate of protein evolution. We used posterior probability to analyze the functional disproportionation of four TaMATE protein subfamilies. The results showed that there was strong functional disproportionation among type I and type II among TaMATE protein subfamilies. Moreover, 72 type II functional disproportionation sites were significantly more than 18 in type I (Figure S1), indicating that the changes in the physicochemical properties of amino acids played a leading role in the differentiation of TaMATE proteins. Among them, 17 functional disproportionation sites shown in Figure 5A were simultaneously involved in type I and type II functional disproportionation, and 16 functional divergence sites were located on the helix. Our results suggest that these sites might play key roles in TaMATE domain differentiation and contribute to forming membrane protein complexes.

Coevolution plays an important role in the evolution of plant species. The identification of coevolutionary sites in a protein family at the molecular level was of great significance for the functional annotation, including the possible interaction between amino acid sites, the interaction between proteins, and the mechanism of adaptation to time changes. In this study, 12 coevolutionary amino acid sites were identified by CAPS, distance-sensitive coevolutionary analysis software for amino acids (Figure 5B). Six sites were located in α-helices and others in coil, but most of them were distributed on the surface of structures, which could benefit to improve the interactions between proteins.

### 2.6. Analysis of Promoter Compositions in TaMATE Genes

The structure of the promoter is very important to the expression of *MATE* genes. PlantCARE was used to analyze the promoter compositions of *TaMATE* genes and eight categories of *cis*-element were identified, including Al resistance elements, light responsive elements, development related elements, hormone responsive elements, environmental stress-related elements, promoter related elements, site-binding related elements and other elements (Figure S2 and Table S3). In these elements, LTR, WUN-motif, GC-motif, ARE, TC-rich repeats and MBS are involved in the low temperature treatment, mechanical damage, hypoxia-inducible, anaerobic induction, stress defense and drought-induced [35,36,37]. In addition, 14 categories of hormone responsive elements were identified, including P-box, TCA-element, GARE-motif, TGA-element, TATC-box, AuxRR-core, ERE, TGACG-motif, CGTCA-motif, and ABRE, etc. [38,39].

In the four subfamilies of TaMATE proteins, each Group II member contained more than one copy of these *cis*-elements that respond to a variety of environmental stresses. Group I and Group IV also had more than one copy of these *cis*-elements that were mainly involved in response to drought and anaerobic conditions. Group III family members contained four kinds of environmental stress response elements, mainly participating in drought stress. This suggests that wheat *MATE* family genes have undergone functional differentiation in response to different environmental stresses.

The important *cis*-acting element, GGN(T/g/a/C)V(C/A/g)S(C/G), was identified as the DNA-binding sequence of ART1 (Al resistance transcription factor 1), which belongs to a C_2_H_2_-type zinc-finger transcription factor and regulates the expression of 31 genes (including *MATE*) to confer Al tolerance in rice. Receptors, such as F-box proteins, would participate in the ART1 modification for balancing Al resistance [40]. In this study, we found that the Al resistance element GGNVS was present in all *TaMATE* genes with different numbers in four subfamilies (Figure S2 and Table S3). In particular, *TaMATE74*, *TaMATE85* and *TaMATE93* genes from Group IV contained 28, 24 and 48 GGNVS elements, respectively. These *TaMATE* genes could play an important role in resistance to Al toxicity.

### 2.7. Transcriptional Expression Profiling of TaMATE Genes in Different Organs and in Response to Abiotic Stresses

The transcriptional expression profiling of *TaMATE* genes was analysed in the root, stem, flag leaf, spike and grain in different developmental stages by using the publicly available transcriptome data. Of the 211 *TaMATE* genes, 138 genes had the expression data and their expression patterns in different organs were shown in Figure S3. Most of the *TaMATE* genes had clear differential expression in different organs, and some of them exhibited highly tissue-specific expression, including roots, stems, leaves and spikes. For example, *TaMATE49*, *TaMATE100*, and *TaMATE114* in Group I had a higher expression level in the root (Figure S3A), while most of the Group II members showed a higher expression in both root and flag (Figure S3B). Five *TaMATE* genes (*TaMATE24*, *TaMATE32*, *TaMATE39*, *TaMATE123* and *TaMATE137*) in Group III are highly expressed in the root while three genes (*TaMATE73*, *TaMATE84* and *TaMATE92*) displayed a higher expression level in leaf (Figure S3C). Six *TaMATE* genes from Group IV (*TaMATE4*, *TaMATE9*, *TaMATE15*, *TaMATE74*, *TaMATE85* and *TaMATE93*) had a higher expression level in both root and leaf (Figure S3D).

**Figure 6:**
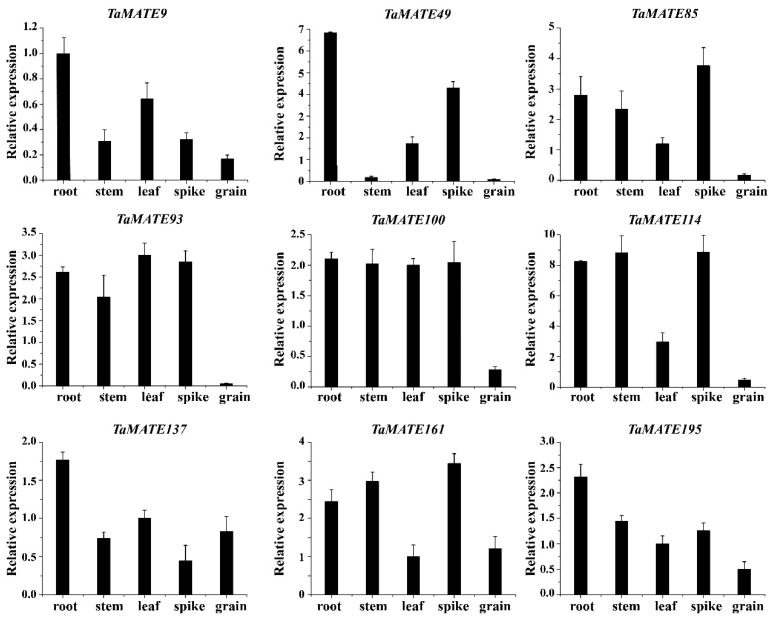
qRT-PCR expression analysis of 9 *TaMATE* genes in root, stem, leaf, spike and grain from Zhongmai 175. Nine *TaMATE* genes included *TaMATE9*, *TaMATE49*, *TaMATE85*, *TaMATE93*, *TaMATE100*, *TaMATE114*, *TaMATE137, TaMATE161* and *TaMATE195*. Error bar represents Sd.

To further verify the reliability of *TaMATE* genes in different organs, we selected nine representative *TaMATE* genes from four subfamilies to perform qRT-PCR analysis (Figure 6), including *TaMATE9*, *TaMATE49*, *TaMATE85*, *TaMATE93*, *TaMATE100*, *TaMATE114*, *TaMATE137*, *TaMATE161* and *TaMATE195*. Their specific primer sequences were listed in Table S4. The results showed that these *TaMATE* genes could express in five organs with different expression levels, indicating their constitutive expression characteristics. All nine *TaMATE* genes displayed an observably high expression level in the root, of which *TaMATE9*, *TaMATE93* and *TaMATE100* were highly expressed in leaf and *TaMATE85*, *TaMATE93*, *TaMATE100*, *TaMATE114* and *TaMATE161* had a higher expression level in spike. These results had a high consistency with transcriptome data (Figure 6 and Figure S3).

The transcription expression profiling of 79 *TaMATE* genes with RNA-seq data in response to abiotic stresses was investigated, including heat stress (1 and 6 h), drought stress (1 and 6 h) and PEG treatment (2 and 12 h). The results showed that *TaMATE* genes from different subfamilies displayed markedly different expression patterns in response to various stresses (Figure S4A–D). For example, *TaMATE100* and *TaMATE114* from Group I and TaMATE161 from Group II had a higher expression level under heat stress.

### 2.8. Transcription Expression Analysis of TaMATE Genes under Aluminum Stress

**Figure 7:**
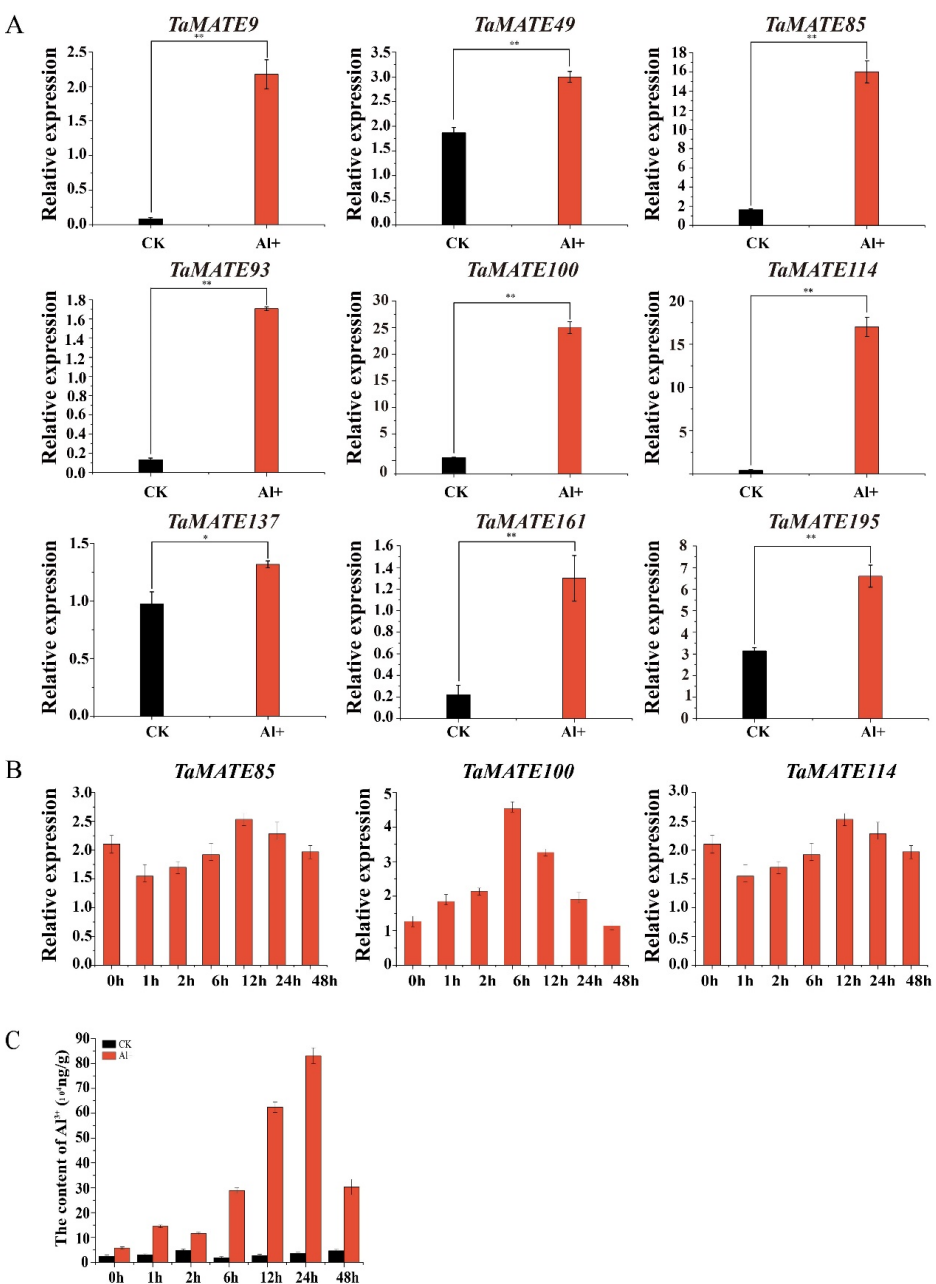
Transcription expression analysis of nine *TaMATE* genes in root and aluminum content changes from Zhongmai 175 root tips under aluminum (Al^3+^) stress. (**A**) Transcription analysis of 9 *TaMATE* genes in root under aluminum stress. Nine *TaMATE* genes included *TaMATE9*, *TaMATE49*, *TaMATE85*, *TaMATE93*, *TaMATE100*, *TaMATE114*, *TaMATE161, TaMATE137* and *TaMATE195*. (**B**) Dynamic expression of three *TaMATE* genes under Al^3+^ stress, three *TaMATE* genes included *TaMATE85*, *TaMATE100* and *TaMATE114*. (**C**) Aluminum content changes of Zhongmai 175 root tips under different times of aluminum stress measured by ICP-MS. *: 0.01 < *p* < 0.05, **: *p* < 0.01; the resulting mean values were presented as relative units. Error bar represents Sd.

*TaMATE* family members have been found to participate in Al tolerance by facilitating citrate efflux in plants. qRT-PCR was used to further reveal the transcription expression patterns of nine *TaMATE* genes in Figure 7 under Al stress. The results indicated that all genes displayed a significantly regulated expression in response to one day treatment with AlCl_3_ (Figure 7A). The dynamic expression profiling of *TaMATE85*, *TaMATE100* and *TaMATE114* under Al stress showed that they had a similar expression pattern, and generally reached the highest expression level at 12 or 6 h (Figure 7B).

To understand the dynamic changes of aluminum content under AlCl_3_ stress in the root, ICP-MS was used to measure Al^3+^ content in Zhongmai 175 root tips under different stress times. The results showed that the accumulation of Al^3+^ in the root tip was significantly increased under Al stress. The highest Al^3+^ content after 24 h treatment of AlCl_3_ reached to 8.31 × 10^5^ ng/g, about 23 times of the control group (Figure 7C). These results were well consistent with the dynamic changes of *TaMATE85*, *TaMATE100* and *TaMATE114* genes under Al stress.

### 2.9. Molecular Docking of the Citrate Binding Sites in TaMATE Proteins

**Figure 8:**
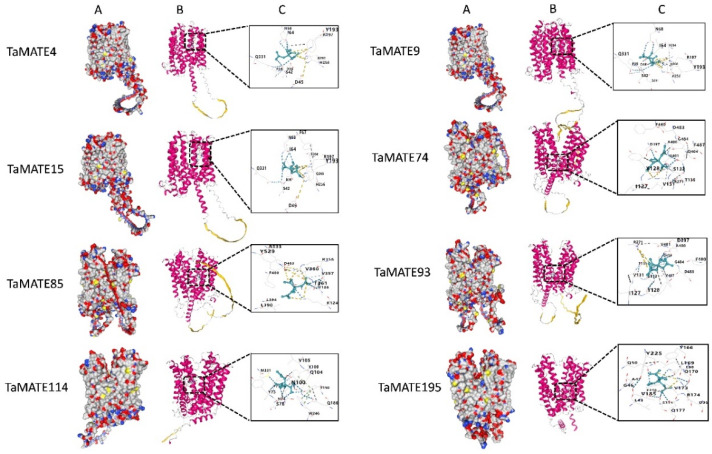
The docking modes of citrate in TaMATE4, TaMATE9, TaMATE15, TaMATE74, TaMATE85, TaMATE93, TaMATE114 and TaMATE195 transporters. (**A**) The binding model of citrate with TaMATEs. (**B**) The binding poses of citrate in different MATE transporters. (**C**) The binding site of citrate in different TaMATE transporters. The model building of 3D structure in wheat MATE protein by AlphaFold.

Transport studies provided concrete evidence in the plant that citrate was transported by MATE transporter under Al stress [28]. To explore the binding poses of TaMATE to citrate, AlphaFold modelling was used for 3D structure prediction of TaMATE proteins, and the molecular docking of the citrate binding residues was performed by CB-dock which is considered the best conformation [41]. Eight representative TaMATE proteins were selected to identify the interactions between citrate and amino acid residues, including TaMATE4, TaMATE9, TaMATE15, TaMATE74, TaMATE85 and TaMATE93 from the Group IV subfamily, TaMATE114 from Group I subfamily, and TaMATE195 from Group II subfamily (Table 1). These protein genes displayed a higher expression level in the roots of wheat (Figure S3 and Figure 7). Among the multiple binding sites predicted with Sitemap, the best sites were selected based on the site score. The results showed that the selected sites were located at the central cavity positioned between the N and C domains of TaMATE transporters (Figure 8A,B).

It is known that the conserved domain of citrate exuding motif (CEM) was present in MATE proteins such as AtFRD3, OsFRDL3, ZmMATE1 and TaMATE1b [28]. When CEM is absent, SbMATE in *Sorghum bicolor* would lose organic cation transport ability [13]. In this study, we found the conserved CEM was located in TaMATE4, TaMATE9, TaMATE15, TaMATE74, TaMATE85 and TaMATE93 with 11, 13, 12, 14, 11 and 15 citrate-binding sites, respectively (Figure 8C). Interestingly, 12 amino acid residues (Asp38, Ser42, Asp 45, IIe64, Phe67, Asn68, Tyr193, Arg197, Val204, His256, Gln260 and Gln331) in CEM were found to bind citrate in TaMATE4, TaMATE9, and TaMATE15. In addition, the conserved Tyr128 residue occurred in TaMATE74, TaMATE85 and TaMATE93, while five residues (IIe127, Tyr128, Val131, Ser132 and Thr135) were present in both TaMATE74 and TaMATE93 (Table 1). These amino acid residues could play important roles in the citrate binding and transportation under Al stress.

In addition, a previous study found that *ZmMATE2* was a major Al-tolerant QTL without a CEM domain but showed an upregulated expression under Al exposure [42]. It should be noticed that although the complete CEM in TaMATE114 and TaMATE195 was absent, the molecular docking showed that they could bind with citrate, and 12 and 15 citrate binding amino acid residues were detected, respectively (Figure 8C). It is possible that these amino acid residues have potential functions for citrate binding and Al detoxification.

It is known that MATEs act as a citrate transporter mediating citrate flux into the xylem, which would facilitate citrate efflux into the rhizosphere to form Al-citrate complexes and chelate Al^3+^, thereby reducing Al toxicity [43]. In Arabidopsis, the mutant of *AtMATE* led to exuding less citrate under Al stress [44]. In rice, the known MATE protein OsFRDL4 could transfer the citrate from the root to the soil at high Al treatment [45,46]. Similarly, GsMATE in soybean and HvMATE in barley could release the citrate from the root pericycle cells to the soil, then chelate the Al^3+^ to detoxify aluminium and adapt the acid soils [47,48]. Meanwhile, along with the Al^3+^ into the root cell under Al stress, Al-citrate complexes could be formed, which might be transported to leaf cells by MATEs such as FeMATE2 in buckwheat and deposited in the vacuole through Golgi [49].

### 2.10. A Putative Transportation Pathway of TaMATE Transporters Resistant to Al Stress in Wheat

**Figure 9:**
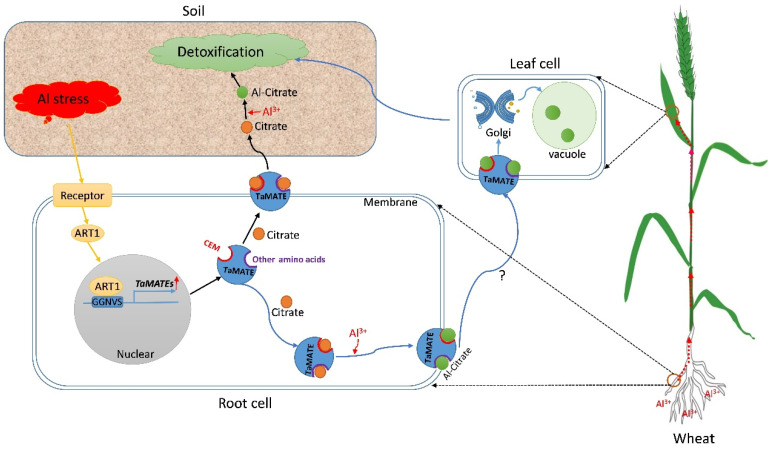
Putative regulatory network of TaMATEs under the treatment of Al^3+^ in the wheat. ART1, Al resistance transcription factor; CEM, citrate exuding motif; *TaMATEs* represent the upregulation of *TaMATE* genes; represents the possible pathway for Al-citrate transfer by TaMATEs; represents Al-citrate transferred from root to leaf; represents the citrate; represents Al-citrate.

Here we proposed a putative transportation pathway of TaMATE transporters resistant to Al stress in wheat according to this study and previous reports (Figure 9). When subjected to Al stress, receptor proteins perceived the external signal and activate ART1, then the ART1 combined with the GGNVS *cis*-element in the upstream of the *TaMATE* genes coding region to enhance *TaMATEs* expression, particularly in the roots and leaves. Subsequently, TaMATEs bond citrate by CEM and other amino acid sites to secrete citrate from root tips to the soil, and then nontoxic Al-citrate complexes in soil were formed to chelate Al and detoxify Al in the wheat rhizosphere. Meanwhile, once Al^3+^ entered into the root cells, Al-citrate complexes could be formed, which might be transported to leaf cells by TaMATEs and then deposited in vacuoles by Golgi transfer system, thereby protecting wheat plants from Al toxicity.

## 3. Material and Methods

### 3.1. Genome-Wide Identification of Wheat MATE Family Genes

Based on the published database, we downloaded 56 and 45 *MATE* gene family members in *Arabidopsis thaliana* and *Oryza sativa*, respectively. Then, their protein sequences were used as seed sequences to perform BlastP and search in WheatOmics (http://202.194.139.32/, accessed on 1 January 2022) and the Ensembl Plants database (http://plants.ensembl.org/Triticum_aestivum/Info/Index, IWGSC RefSeq v2.1, version 44, accessed on 1 January 2022) and obtain wheat MATE protein sequences and the threshold E-value was set to ≤1 × 10^−5^. Then, the subjected sequences were fed into the SMART (Simple Modular Architecture Research Tool) (http://smart.embl-heidelberg.de/, accessed on 2 January 2022) website and Pfam (http://pfam.xfam.org/, accessed on 2 January 2022) database one by one to detect whether the candidate sequence contains a conserved MATE protein domain. Finally, all MATE protein sequences, their corresponding CDS sequences and genome sequences identified in wheat were used for subsequent analysis.

### 3.2. Phylogenetic and Structure Analysis

The MATE coding sequences were obtained from the downloaded data (ftp://ftp.ensemblgenomes.org/pub/plants/release-42/fasta) in *Arabidopsis thaliana* and *Oryza sativa*. *MUSCLE* software (http://www.drive5.com/muscle/manual/, accessed on 5 January 2022) was used for amino acid (aa) alignments. A phylogenetic tree was constructed by MEGA 6.0 software (Koichiro Tamura, Tokyo, Japan) with the Bayesian method and 1000 bootstrap tests. Gene structure was analysed by using TBtools. The Multiple Em for Motif Elicitation v 4.11.4 (MEME) was used to identify conserved motifs, and the maximum number of motifs set at 10.

### 3.3. Chromosomal Location and Collinearity Analysis

The chromosome location of each *TaMATE* gene was determined by IWGSC RefSeq v2.1 (cv. Chinese_Spring) by using Blast programs (https://blast.ncbi.nlm.nih.gov/Blast.cgi, accessed on 3 January 2022). Their locations were mapped by the MapInspect tool (http://mapinspect.software.informer.com/, accessed on 5 January 2022). TBtools (v1.077) was used to do the duplication analysis of *TaMATEs* in wheat.

### 3.4. Subcellular Localisation of TaMATE Proteins

The subcellular localisation of TaMATEs was predicated by the websites of WoLF PSORT (https://wolfpsort.hgc.jp/, accessed on 5 January 2022), Plant-mPLoc (http://www.csbio.sjtu.edu.cn/bioinf/plant-multi/, accessed on 5 January 2022), CELLO v.2.5 (http://cello.life.nctu.edu.tw/, accessed on 5 January 2022), UniProtKB (https://www.uniprot.org/help/uniprotkb/, accessed on 5 January 2022) and TargetP-2.0 (http://www.cbs.dtu.dk/services/TargetP/, accessed on 5 January 2022). Then, a further subcellular localisation assay was performed via wheat mesophyll protoplast transformation based on the reported method [50].

### 3.5. Three-Dimensional (3D) Structure and Molecular Evolution Analysis of TaMATE Proteins

The 3D structure of TaMATE proteins was constructed using the AlphaFold [32,33,34]. Then, editing was performed by Pymol software (version 1.7.4 Schrödinger, Warren Lyford DeLano, New York City, NY, USA). TaMATEs protein topology was predicted by Protter (http://wlab.ethz.ch/protter, accessed on 2 February 2022). Coevolution sites were identified by Coevolution Analysis Protein Sequences (CAPS) software. DIVERGE v2.0 software package combined with posterior probability analysis was used to analyze the function disproportionation between different subfamilies of the *TaMATE* gene family.

### 3.6. Identification of the Cis-Acting Elements in the TaMATE Genes

The members of *TaMATE* genes were unified into IWGSC gene ID, and *cis-acting* elements in the 1500 bp upstream promoter regions of the identified *TaMATE* genes were identified via PlantCARE (http://bioinformatics.psb.ugent.be/webtools/plantcare/html/, accessed on 15 February 2022). All of these sequences were used to identify the *cis*-acting elements by the recently released *Triticum aestivum* genome database (IWGSC RefSeqv2.1) with a coverage rate of 94% from GRAMENE (http://ensembl.gramene.org/, accessed on 10 February 2022).

### 3.7. TaMATE Gene Expression Analysis by RNA-Seq Data

The RNA-seq data of the *TaMATE* genes were downloaded from the expVIP website (http://www.wheat-expression.com/, accessed on 2 February 2022) [51] and cluster analysis was performed by TBtools.

### 3.8. Plant Materials and Al Stress Treatments

The seedlings of Elite Chinese wheat cultivar Zhongmai 175 were cultivated into two and a half leaf stages according to the culture conditions [52]. Then seedlings were treated with the conditions of normal and Al stress with 50 μM AlCl_3_. The samples from AlCl_3_ were collected at 0, 1, 2, 6, 12, 24, 48 (recover) h, and other treated seedlings were harvested at 2 h. Samples were collected from three biological replicates and then frozen in liquid nitrogen immediately.

### 3.9. Measurement of Total Al Content in Root

Al-treated and untreated wheat roots were dried for 3 d at 55 °C and then put into a digestion tank. Pre-digesting was conducted by adding 5 mL 65% HNO_3_ (Suprapur, Merck, Darmstadt, Germany) and 2 mL H_2_O_2_ (Suprapur, Merck, Darmstadt, Germany) to the digestion tank for 40 min at room temperature. The samples were digested by a microwave digestion instrument (MARS, CEM Corporation, Matthews, NC, USA) for 0, 1, 2, 6, 12, 24, and 48 h. Al content (μg/g DW) was detected by using inductively coupled mass spectrometry (ICP-MS, ELAN DRC-e, PerkinElmer, Waltham, MA, USA) based on the method in the previous report [53].

### 3.10. Total RNA Extraction and qRT-PCR

Total RNA was isolated from wheat samples by using TRIzol reagent (Invitrogen, Waltham, MA, USA) based on the manufacturers’ instructions. Qrt-RCR was carried out using an Eco Real-time PCR system (Illumina, Los Angeles, CA, USA) with SYBR^®^ Premix Ex TaqTM (TaKaRa, Shiga, Japan). The primers were designed by Primer premier 5.0. Wheat Ubiquitin was used as the reference control. The relative expression levels of *TaMATE* genes were analysed with the comparative threshold cycle method 2^−ΔΔCT^ [54].

### 3.11. Molecular Docking and Binding Site Analysis

AlphaFold was used to predict the 3D models of TaMATE, and the ligand structure was identified in the NCBI (https://pubchem.ncbi.nlm.nih.gov/, accessed on 1 February 2022). To understand the interactions of the selected citrate with different TaMATE transporters, molecular docking was performed with CB-Dock (http://cao.labshare.cn/cb-dock/, accessed on 1 February 2022) [41]. A more negative docking score indicates the better binding strength of a ligand. Then, MSA (Multiple Sequence Alignment) was used to check the conserved binding site in CEM.

## 4. Conclusions

Genome-wide analysis identified 211 *TaMATE* genes in wheat, which were classified into four subfamilies, respectively named Group I, II, III and IV. The *TaMATE* genes in the same subfamily had similar motif and intron/exon compositions, but those in different subfamilies showed clear differences. The segmental and tandem duplication played main roles in the amplification of wheat *MATE* genes, and Type II functional disproportionation among subfamilies was largely responsible for the differentiation of wheat *MATE* genes. The promoter region of *TaMATE* genes contained abundant Al resistance and environmental stress-related *cis*-acting elements that enhance the high expression of *TaMATE* genes in roots and in response to Al stress. The 3D structure modelling by AlphaFold and molecular docking by CB-dock indicated that plasmalemma-localised TaMATE proteins could combine with citrate via amino acid residues in CEM and other sites, and then release citrate out of the root cells to chelate aluminium, thereby alleviating Al toxicity. On the other hand, the citrate aluminium complex formed in plants might be transported to leaves by TaMATEs and then deposited in vacuoles to reduce Al toxicity. A putative transportation pathway of TaMATE transporters resistant to Al stress in wheat was put forward, which provides new insights into the molecular mechanisms of the plant *MATE* gene family involved in Al tolerance. Our results demonstrate that *TaMATE* genes have potential application values for the genetic improvement of crop Al tolerance.

## Figures and Tables

**Table 1:** The amino acid sites binding citrate in different TaMATE transporters.

TaMATEs	Site with Citrate *
TaMATE4	Asp (38), Pro (39), Ser (42), Asp (45), IIe (64), Asn (68), Tyr (193), Arg (197), His (256), Gln (260), Gln(331)
TaMATE9	Asp(38), Pro(39), Ser (42), IIe (64), Asn (68), Tyr (193), Arg (197), Val (204), Asp (231), His (256), Gln (260), Gln (331)
TaMATE15	Asp (38), Ser (42), Asp (45), IIe (64), Phe (67), Asn (68), Tyr (193), Arg (197), Val (204), His (256), Gln (260), Gln (331)
TaMATE74	IIe (127), Tyr (128), Val (131), Ser (132), Thr (135), Arg (271), Asp (397), Ala (400), Val (401), Gln (404), Phe (480), Asp (483), Gly (484), Phe (487)
TaMATE85	Lys (124), Tyr (128), Arg (356), Val (357), Val (360), Thr (361), Leu (390), Leu (394), Phe (480), Tyr (529), Arg (533)
TaMATE93	IIe (127), Tyr (128), Val (131), Ser (132), Thr (135), Arg (271), Asp (397), Ala (400), Val (401), Gln (404), Phe (480), Asp (483), Gly (484), Phe (487)
TaMATE114	Tyr (71), Asn (74), Tyr (75), Ser (78), Asn (100), Gln (104), Val (105), Tyr (108), Gln (186), Tyr (190), Trp (246), Met (331)
TaMATE195	Leu (43), Gly(46), Ala(47), Gln(50), Leu(88), Asp(95), Tyr(166), Leu(169), Gln(170), Val(173), Arg(174), Val(185), Tyr(225), Phe(310), Ser(314)

* Red marked amino acid site represent the amino acid in CEM (citrate exuding motif).

## Data Availability

Not applicable.

## Supplementary Materials

The following supporting information can be downloaded at: https://www.mdpi.com/article/10.3390/ijms23084418/s1.

## Abbreviations

ALMT	Aluminium activated malate transporter
ART1	Al resistance transporter factor1
CEM	Citrate exuding motif
ICP-MS	Inductively couple mass spectrometry
MATE	Multidrug and toxin efflux
qRT-PCR	Quantitative real-time polymerase chain reaction
SMART	Simple Modular Architecture Research Tool
