# Supplementary material for: Genome-Wide Identification and Characterisation of Wheat MATE Genes Reveals Their Roles in Aluminium Tolerance
Source: Int J Mol Sci. 2022 Apr 16;23(8):4418. doi: 10.3390/ijms23084418 (PMC9030646; doi:10.3390/ijms23084418)
Supplement: Supplementary file 1 [file ijms-23-04418-s001.zip › Figure S1-S4.pdf]

Genome-Wide Identification and Characterisation of Wheat *MATE* Genes Reveals Their Roles in Aluminium Tolerance

Wenjing Duan †, Fengkun Lu †, Yue Cui †, Junwei Zhang, Xuan Du, Yingkao Hu * and Yueming Yan *

Beijing Key Laboratory of Plant Gene Resources and Biotechnology for Carbon Reduction and Environmental Improvement, College of Life Science, Capital Normal University, Beijing 100048, China;
duanwenjings@163.com (W.D.); lfksiwuliu@163.com (F.L.); cy9551@yeah.net (Y.C.); zhangjunwei_22@163.com (J.Z.); duxuan503@163.com (X.D.)
* Correspondence: yingkaohu@cnu.edu.cn (Y.H.), yanym@cnu.edu.cn (Y.Y.)
† These authors contributed equally to this work.

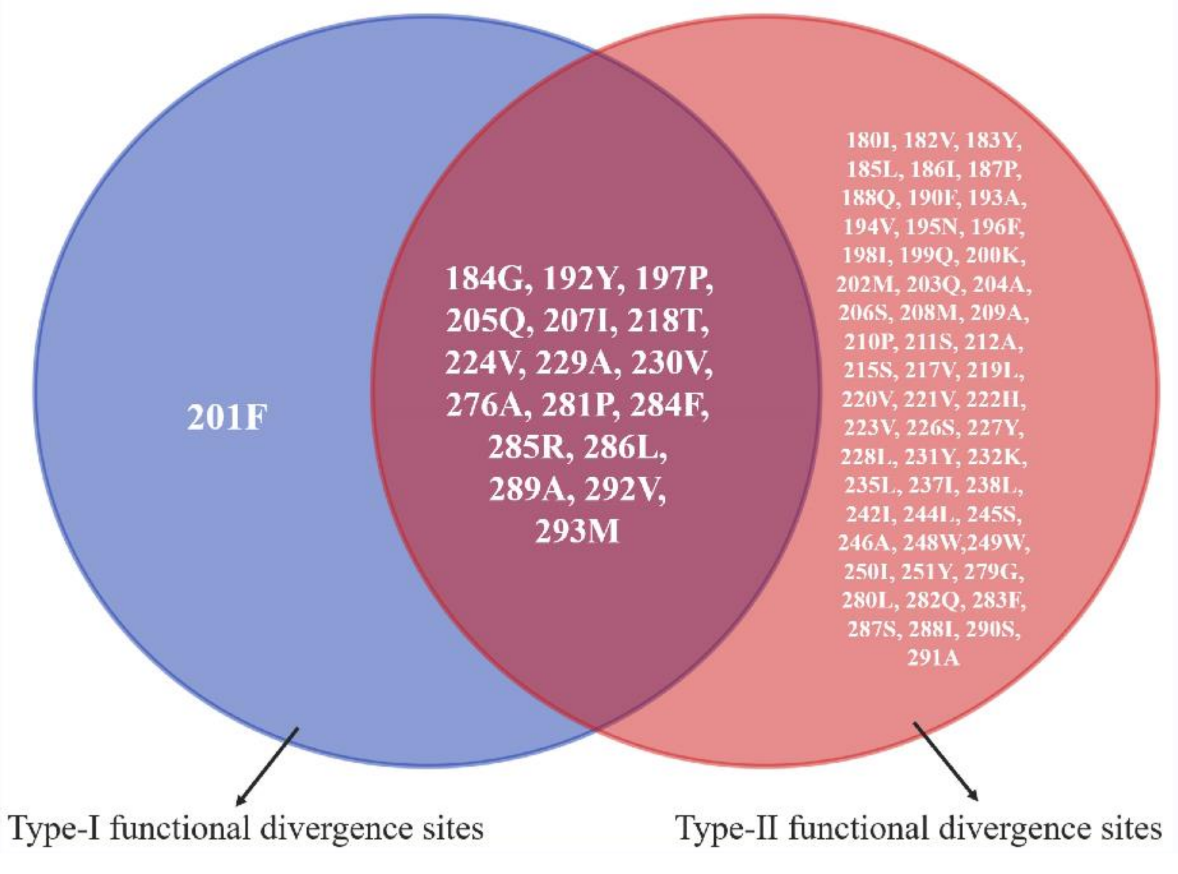

Figure S1. Crucial amino acid sites of type-I and type-II functional divergence.

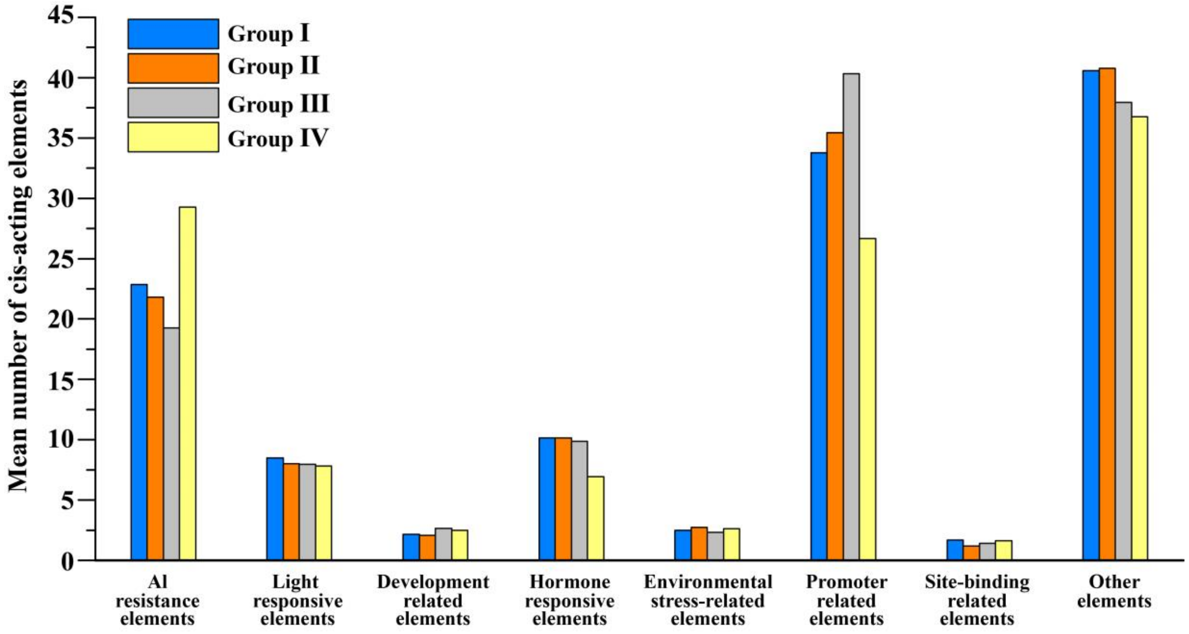

Figure S2. Promoter compositions of MATE family genes in common wheat. Different colour blocks represent different TaMATE subfamilies.

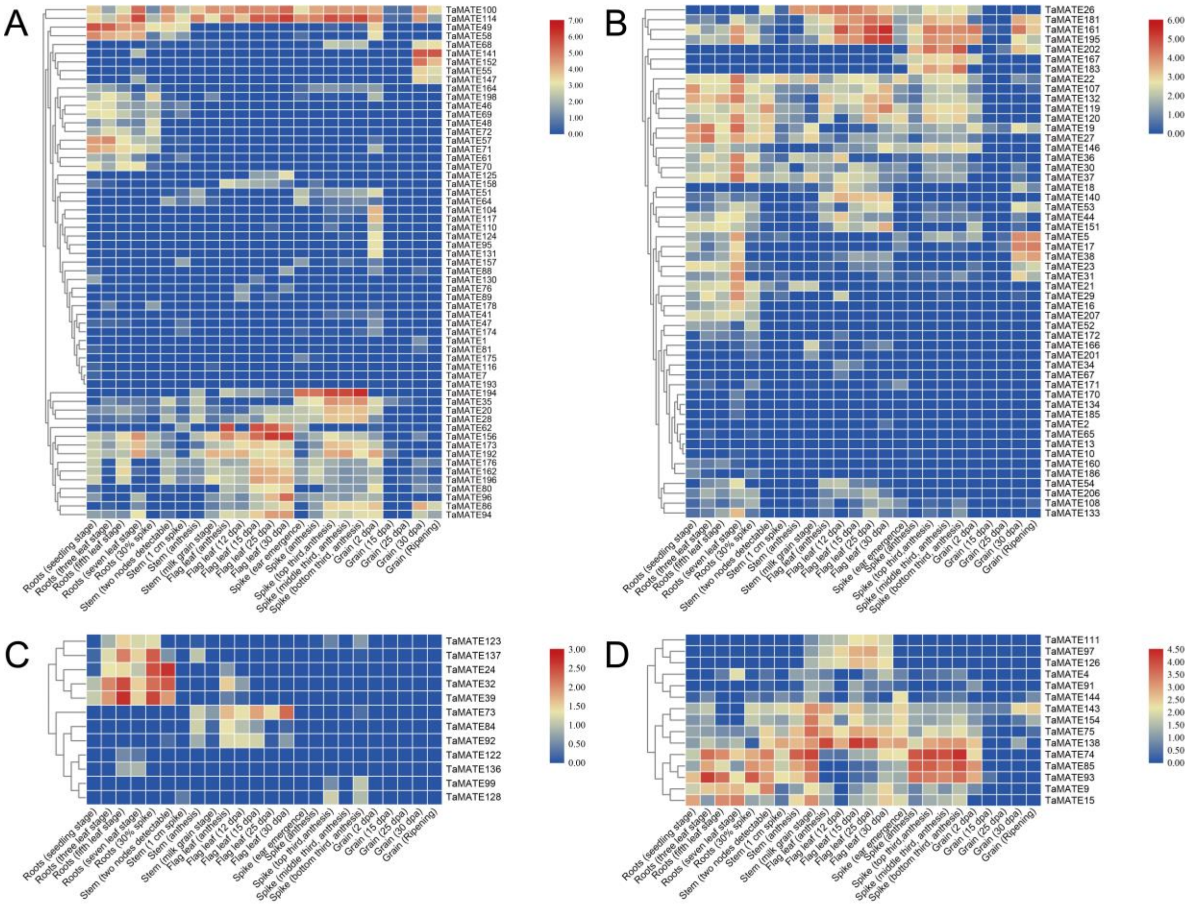

Figure S3. Expression profile of *TaMATE* genes in five different tissues of wheat. The normalised expression values of genes in roots (seedling stage, three leaf stage, fifth leaf stage, seven leaf stage, 30% spike), stem (1 cm spike, anthesis, milk grain stage), flag leaf (12, 15, 25, 30 dpa; dpa, days post anthesis), spike (ear emergence, anthesis, top third anthesis, mild third anthesis, bottom third anthesis) and grain (2, 15, 25, 30 dpa and ripening) were extracted from the analysed transcriptome data. The heat map of 138 *TaMATE* genes was generated with Genesis software. The expression values (FPKM+1) of *TaMATE* genes were transformed by log2 and mean centered. A, B, C, and D represent the TaMATE genes in Group I, Group II, Group III and Group IV, respectively.

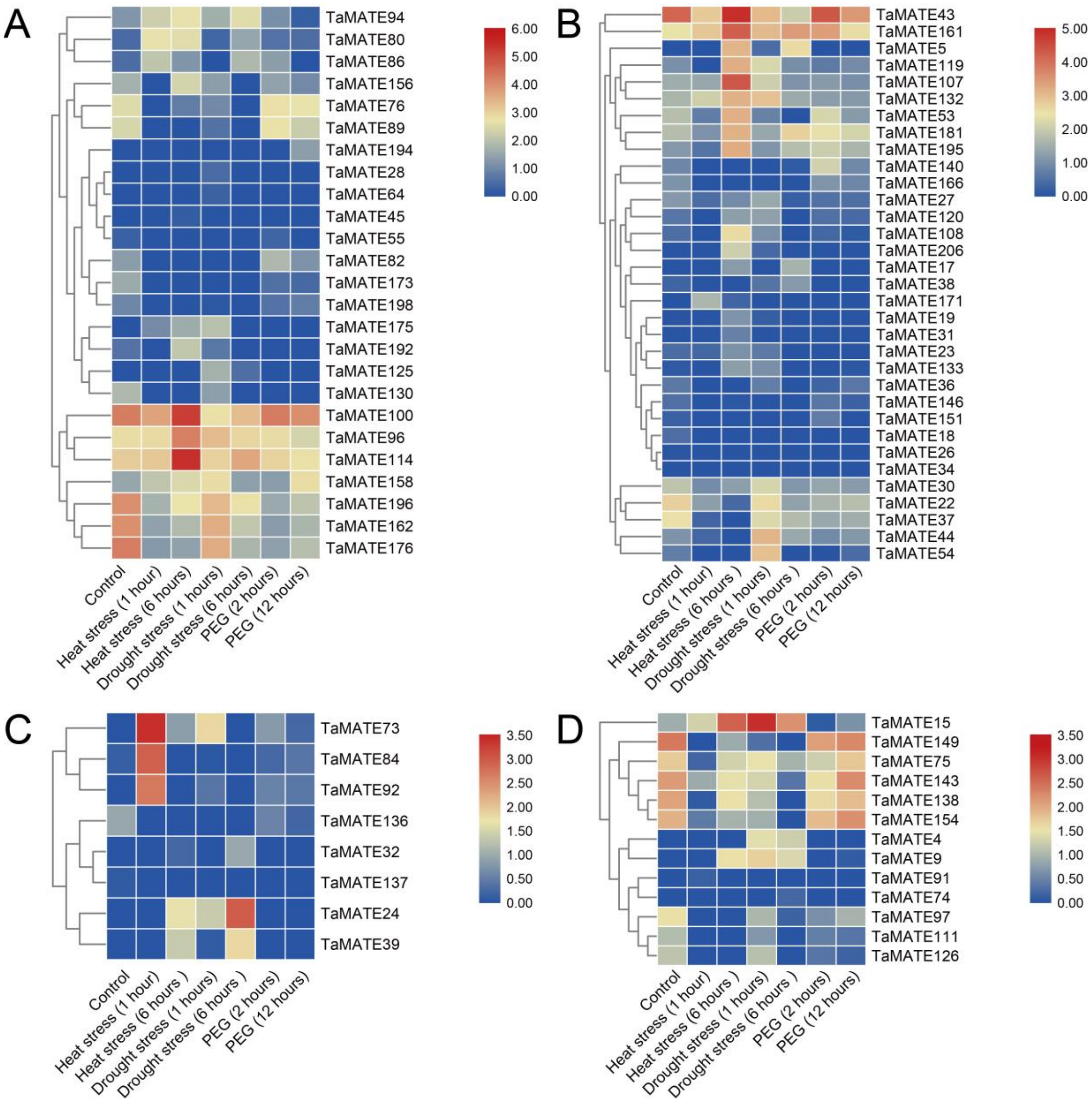

Figure S4: Expression patterns of *TaMATE* genes under heat stress (1 and 6 h), drought stress (1 and 6 h) and PEG (2 and 12 h) treatment. The heat map of 79 TaMATE genes was generated with Genesis software. The expression values (FPKM+1) of *TaMATE* genes were transformed by log2 and mean centered. A, B, C, and D represent the TaMATE genes in Group I, Group II, Group III, and Group IV, respectively.
